# Knowledge, Attitude, and Practice of Artificial Intelligence (AI) among Rheumatology Professionals: An Online Cross-Sectional Survey

**DOI:** 10.31138/mjr.250924.koc

**Published:** 2025-01-23

**Authors:** Sabarinath Mahadevan, Mahabaleshwar Mamadapur, Rahul Bisaralli, Anna C. Das, Pavan Kumar MR, Veerendra Arakalavadi Guruswamy, Rajath K. Bharadwaj

**Affiliations:** 1 Institute of Rheumatology, Madras Medical College, Tamil Nadu, India;; 2 Department of Clinical Immunology and Rheumatology, JSS Medical College and Hospital, JSS Academy of Higher Research, Mysore, Karnataka, India;; 3 Department of Clinical Immunology and Rheumatology, SDM College of Medical Sciences and Hospital, Dharwad, Karnataka, India;; 4 Department of Clinical Immunology and Rheumatology, St John’s Medical College and Hospital, St John’s National Academy of Medical Sciences, Bangalore, India;; 5 Department of Pharmacy Practice, JSS College of Pharmacy Mysuru, JSS AHER, Mysuru, India

**Keywords:** artificial intelligence, machine learning, deep learning, knowledge attitude and practice, rheumatology, surveys and questionnaires

## Abstract

**Introduction:**

Artificial intelligence (AI), as a significant technological advancement, aims to simulate and automate human intelligence.

**Aim/Objective:**

To assess the knowledge, attitude, and practice of artificial intelligence among rheumatology professionals in India.

**Methods:**

We invited rheumatology professionals to complete an online survey from June to September 2024. Online surveys were circulated in social media and emails. A descriptive analysis was conducted to compare whether survey responses varied between respondents who received training in AI earlier, consultants, and residents in rheumatology.

**Result:**

The survey included 173 participants, mostly from private hospitals. The response rate was 11.53%. While awareness of AI was high (98.3%), only 54.3% understood specific terminology such as machine learning. Although 81.5% believed AI could enhance patient care, only 33.5% regularly used AI tools in clinical practice. A substantial knowledge gap exists, and there is a need for formal training sessions in Artificial Intelligence among rheumatology professionals. The main obstacles to applying AI were patient data security concerns (64.2%), potential ethical and human implications of using AI in medicine (25.4%), and insufficient training (3.5%). Half feared losing their expertise, and AI had a difficult learning curve.

**Conclusion:**

The results indicate a substantial knowledge gap and the need for formal training sessions in Artificial Intelligence among rheumatology professionals.

## INTRODUCTION

Artificial intelligence (AI), as a significant technological advancement, aims to simulate and automate human intelligence. The advent of ChatGPT and other chatbots has accelerated the awareness and application of AI across various fields. Numerous studies have explored the applications of AI in medicine. In general, AI has shown a promising role in medical education, analysis of hospital records, patient management systems, supporting diagnosis through image mapping, aiding in research by automating new drug discovery, developing prediction models, supporting plugins for medical devices, assisting in patient education, and much more.^1–4^ The digitalisation of health records and the availability of large datasets are poised to revolutionise medical specialities through AI.^5^

As much as AI technology is fascinating and generates high expectations, it is not without challenges. The complexity of underlying principles, such as neural networks and large language models, can be difficult for clinicians to comprehend.^6^ This may challenge clinicians’ knowledge, attitudes, and practices regarding AI. Additionally, the applications and apprehensions regarding AI vary across different medical subspecialties. For example, in specialities like radiology and dermatology, where image mapping is crucial, AI is thought to exceed human limitations due to its more significant memory and data storage capabilities.^7^ As such, KAP regarding AI could not be generalised across the medical subspecialty. Multiple studies have assessed AI’s knowledge, attitudes, and practices (KAP) across specialities like paediatrics, radiology, etc.^8,9^

Rheumatology is a unique speciality that requires clinical insight for diagnosis and management. Laboratory tests in rheumatology have limitations of false positives, false negatives hampering the predictive value of most tests. Consequently, tests done in rheumatology have limited stand-alone diagnostic ability. Also, most criteria commonly used in rheumatology are meant to classify diseases rather than diagnose patients.^10^ The rarity and baseline complexity of many rheumatological diseases also impact the feasibility of controlled trials, often leaving standard body guidelines to emphasise individualised management plans for clinical decision-making. In this speciality, the automation of results and the role of simulated intelligence are particularly intriguing. We aimed to assess the knowledge, attitudes, and practices (KAP) regarding AI among doctors in this subspecialty. Perception checking is essential to understand the KAP gap about AI among rheumatologists, which may pave the way for addressing the concerns specific to this speciality. To the best of our knowledge, no study has yet focused on the KAP of rheumatologists toward AI. In this context, we invited rheumatologists nationwide to participate in this online survey.

## OBJECTIVES

- To assess the level of knowledge and attitudes among Rheumatology practitioners and trainees about AI and its implications.
- To evaluate the understanding of contemporary AI practices.

## METHODS

We conducted a cross-sectional study involving rheumatologists nationwide from June 14 2024, to September 14 2024. We aimed to include rheumatology residents and consultant rheumatologists in teaching and non-teaching hospitals. Residents included doctors who had completed three years of internal medicine or paediatrics and were currently enrolled in the three-year training program for Clinical Immunology and Rheumatology at centres recognised by the National Medical Commission. Consultants included doctors who had completed three years of training in rheumatology and were currently working in teaching or non-teaching hospitals across the country.

We sent questionnaires via email to all members of the national rheumatology forum. Participation was announced as anonymous and optional. We followed the CHERRIES (Checklist for Reporting Results of Internet E-Surveys) and STROBE (Strengthening the Reporting of Observational Studies in Epidemiology) frameworks statement for reporting (see appendix). There was no age limit for participation. Only one response was allowed from each email ID to avoid duplication. Participation in the study was voluntary. A brief introduction was provided at the beginning of the questionnaire to inform participants about the study objectives and ensure confidentiality. Completing the questionnaire was regarded as valid consent. Respondents were allowed to go back and forth to change their responses until the end of the survey.

We used a convenient sample size for this study, utilising a questionnaire developed through Google Forms. The questionnaire aimed to capture demographic data and familiarity with AI and explore knowledge about AI, expected applications, merits, demerits, and potential challenges in medical practice. To the best of our knowledge, a study assessing rheumatology professionals’ knowledge, attitude, and training has yet to be done. The questionnaire was developed after a literature review of similar studies in other sub-specialities like paediatrics, radiology, and dermatology.^8–10^ An expert panel, including four rheumatologists and one Professor of Community Medicine, validated the questionnaire. The questionnaire underwent two revisions. The questionnaire’s reliability was initially pre-tested among local regional rheumatologists representing the target population (n=20). A Cronbach’s alpha of 0.63 was obtained, which is generally acceptable.

Baseline data, including age, expertise level (student or consultant), and working station (non-teaching or teaching), were collected through initial questions (1–5). The survey was structured into three main sections: knowledge (questions 6–8), attitude (questions 9–21), and practice (questions 22–28). We used both closed and open-ended questions. Closed-ended questions were collected on a 5-point Likert scale (agree, rather agree, neutral, rather disagree, and disagree). The answers were then grouped under yes (agree, rather agree), neutral, and no (rather disagree, disagree). Open-ended questions provided multiple options along with an “other” option to include reasoning not listed by the authors. Respondents were allowed to skip questions if they wished. Ethics approval was obtained from the institutional ethics committee.

The responses were compiled in an MS Office Excel sheet. We conducted a descriptive analysis of the baseline data, summarising responses as frequencies and percentages. We assessed the KAP between residents and consultants, those with formal training versus those without, and those in teaching versus non-teaching institutions using Chi-square. A p-value of less than 0.05 was considered significant. Statistical analysis was performed using SPSS software.

## RESULTS

One thousand five hundred rheumatology professionals were invited through mailing lists and social media. A total of 173 rheumatology professionals responded to the survey. The response rate was 11.53%. Due to possible overlap between participants on social media and email, it took time to determine the participation rate.

**Table 1** provides data on the demographic and professional characteristics of the study subjects. The age group of 25–35 comprises the largest portion, representing 43.4% of the total, and the category of 36–45 follows, with 30.6%. 19.1% fell into the 46–60 years group and 6.9% from the above 60 years category. The male population comprises the majority, accounting for 64.7%, and females accounting for 35.3%. Most participants (46.8%) were employed in private or corporate hospitals. 27.2% of rheumatologists were employed in private-sector teaching hospitals, 21.4% in government-sector teaching hospitals, and 3.5% in Central Institutes.

**Table 1.** Subject distribution.

**AGE DISTRIBUTION**		
25–35 years	75	43.4
36–45 years	53	30.6
46–60 years	33	19.1
>60 years	12	6.9
**GENDER**		
Male	112	64.7
Female	61	35.3
**AREA OF PRACTICE**		
Central Institute	6	3.5
Non-Teaching hospital in Govt Sector	2	1.2
Private/corporate hospital	81	46.8
Teaching hospitals in the private sector	47	27.2
Teaching hospitals in the sector	37	21.4
**RHEUMATOLOGY TRAINEE**		
No	124	71.7
Yes	49	28.3

The findings indicated (**Table 2**) that a significant proportion of rheumatologists (98.3%) were aware of AI. In comparison, only 54.3% were acquainted with the specific terminology associated with AI, such as deep learning and machine learning. The majority of our rheumatologists (81.5%) expressed the belief that it could enhance patient care. 20.2% believed it would transform the medical speciality. Only 14.5% had attended formal sessions on using various AI tools. Most rheumatologists (81.5%) hold a favourable view of AI and anticipate a beneficial influence on the field of rheumatology. However, only 33.5% of rheumatologists presently use AI in their regular practice (almost daily/weekly once). Regarding practice, only 15% had attended a formal session on using AI tools, and 36% had interacted with AI via particular or non-specific training.

**Table 2.** Assessment of knowledge, attitude and practice.

		**Rheumatology trainee**				**Total**		**χ2 - Value (P - Value)**	**Odds (CI)**
		**No**		**Yes**					
		**N**	**%**	**N**	**%**	**N**	**%**		
**KNOWLEDGE**									
Are you aware of artificial intelligence AI?	Yes	122	98.4%	48	98.0%	170	98.3%	0.038 (1.000) NS	1.271 (0.113 – 14.343)
	No	2	1.6%	1	2.0%	3	1.7%		
From where did you get to know about AI?	Social media	79	63.7%	26	53.1%	105	60.7%	17.084 (0.0001) S	-
	Colleagues or Friends	5	4.0%	13	26.5%	18	10.4%		
	Academic Forum or Articles	40	32.3%	10	20.4%	50	28.9%		
Have you heard of AI terminologies like deep learning AI, machine learning AI, Generative AI, etc?	Yes	57	46.0%	22	44.9%	79	45.7%	0.016 (1.000) NS	1.044 (0.537 –2.029)
	No	67	54.0%	27	55.1%	94	54.3%		
**ATTITUDE**									
Would AI be a valuable tool in the medical speciality?	Yes	99	79.8%	42	85.7%	141	81.5%	1.003 (0.651) NS	-
	Maybe	24	19.4%	7	14.3%	31	17.9%		
	Neutral	1	0.8%	0	0.0%	1	0.6%		
What are the areas where you think AI would be helpful in medical specialty?	Medical Education	100	80.6%	39	79.6%	139	80.3%	-	-
	Diagnosis and Management of Patients	17	13.7%	2	4.1%	19	11.0%	9.431 (0.016) S	-
	Research and Publication	1	0.8%	4	8.2%	5	2.9%		
	Clerical Work and Documentation of patient record	6	4.8%	4	8.2%	10	5.8%		
What aspect of medical education do you think AI will be useful? (Multiple answers)	Typing articles for publication	74	59.7%	30	61.2%	104	60.1%	2.293 (0.844) NS	-
	Preparing presentations	24	19.4%	12	24.5%	36	20.8%		
	Providing reading materials for students	18	14.5%	7	14.3%	25	14.5%		
	Exams and Evaluation	3	2.4%	0	0.0%	3	1.7%		
Do you think AI will promote better comprehension and learning of complex topics in medicine?	Yes	68	54.8%	30	61.2%	98	56.6%	0.625 (0.755) NS	-
	No	8	6.5%	2	4.1%	10	5.8%		
	Neutral	48	38.7%	17	34.7%	65	37.6%		
Should artificial intelligence be part of the medical curriculum?	Yes	88	71.0%	33	67.3%	121	69.9%	0.438 (0.865) NS	-
	No	6	4.8%	2	4.1%	8	4.6%		
	Neutral	30	24.2%	14	28.6%	44	25.4%		
Do you believe AI technology does threaten your career?	Yes	12	9.7%	4	8.2%	16	9.2%	0.303 (0.867) NS	-
	No	81	65.3%	31	63.3%	112	64.7%		
	Neutral	31	25.0%	14	28.6%	45	26.0%		
Do you believe that AI will provide ethical challenges?	Yes	83	66.9%	33	67.3%	116	67.1%	2.684 (0.270) NS	-
	No	7	5.6%	6	12.2%	13	7.5%		
	Neutral	34	27.4%	10	20.4%	44	25.4%		
Which of the following areas in clinical practice do you think AI may be of use?	Clinical diagnosis and management	65	52.4%	19	38.8%	84	48.6%	7.751 (0.118) NS	-
	Patient counselling, issuing handouts, etc	29	23.4%	12	24.5%	41	23.7%		
	Documentation and Clerical work	28	22.6%	16	32.7%	44	25.4%		
	Calculation of drug dosages, infusions, disease scores, etc	1	0.8%	0	0.0%	1	0.6%		
	Interpretation of multiple laboratory reports and images	0	0.0%	2	4.1%	2	1.2%		
	Make personalised pharmacological prescriptions for individuals	1	0.8%	0	0.0%	1	0.6%		
What are the possible problems AI may lead to in medical research and publication?	Ethically Wrong	61	49.2%	26	53.1%	87	50.3%	1.056 (0.799) NS	-
	Authorship Conflict	31	25.0%	12	24.5%	43	24.9%		
	Misleading Scientific Information / Statistical Manipulation	29	23.4%	9	18.4%	38	22.0%		
	Others	3	2.4%	2	4.1%	5	2.9%		
What are the merits of AI according to you?	Ability to learn and refine its answers	74	59.7%	28	57.1%	102	59.0%	1.223 (0.772) NS	-
	Provide answers within a short timespan	43	34.7%	20	40.8%	63	36.4%		
	Accuracy and relevancy of information	4	3.2%	1	2.0%	5	2.9%		
	Others	3	2.4%	0	0.0%	3	1.7%		
What do you think of the demerits of AI?	Not available in all languages	38	30.6%	14	28.6%	52	30.1%	3.368 (0.490) NS	-
	Can't personalise therapy	47	37.9%	16	32.7%	63	36.4%		
	Ethical issues	26	21.0%	9	18.4%	35	20.2%		
	This may lead to a clinical mishap	11	8.9%	9	18.4%	20	11.6%		
	Others	2	1.6%	1	2.0%	3	1.7%		
What do you think about AI tools in the medical specialty in the future?	Unlikely to influence medical speciality	6	4.8%	1	2.0%	7	4.0%	2.672 (0.642) NS	-
	May influence / likely to stay as an optional use	39	31.5%	16	32.7%	55	31.8%		
	A must in the future/part of the routine in medicine	51	41.1%	20	40.8%	71	41.0%		
	Revolutionalise medical specialty	23	18.5%	12	24.5%	35	20.2%		
	Others	5	4.0%	0	0.0%	5	2.9%		
Would you recommend AI to your colleagues in medicine?	Yes	116	93.5%	48	98.0%	164	94.8%	1.386 (0.448) NS	0.302 (0.037 – 2.481)
	No	8	6.5%	1	2.0%	9	5.2%		
**PRACTICE**									
Have you attended Webinars or Panel discussions on the role of Allied Healthcare?	Yes	57	46.0%	22	44.9%	79	45.7%	0.016 (1.000) NS	1.044 (0.537 – 2.029)
	No	67	54.0%	27	55.1%	94	54.3%		
Have you had any formal sessions on using various AI tools?	Yes	17	13.7%	8	16.3%	25	14.5%	0.195 (0.639) NS	0.814 (0.326 – 2.031)
	No	107	86.3%	41	83.7%	148	85.5%		
Have you ever interacted with AI via particular or non-specific training?	Yes	49	39.5%	20	40.8%	69	39.9%	0.025 (1.000) NS	0.947 (0.483 – 1.859)
	No	75	60.5%	29	59.2%	104	60.1%		
Which of the following AI tools have you ever used?	None	100	80.6%	42	85.7%	142	82.1%	1.260 (0.922) NS	-
	ChatGPT	19	15.3%	6	12.2%	25	14.5%		
	Google Assistant	1	0.8%	0	0.0%	1	0.6%		
	Gemini	1	0.8%	0	0.0%	1	0.6%		
	Quillbot	3	2.4%	1	2.0%	4	2.3%		
	Cortana (Microsoft)	-	-	-	-	-	-		
How often do you use AI?	Routinely	35	28.3%	12	24.5%	47	27.2%	0.316 (0.595) NS	0.820 (0.410 – 1.639)
	Rarely	5	4.0%	6	12.2%	11	6.3%		
	Never	84	67.7%	31	63.3%	115	66.5%		
What are the obstacles to the implementation of AI?	Patient data security	81	65.3%	30	61.2%	111	64.2%	3.465 (0.657) NS	-
	Threat to the ethical and human aspects of medicine	32	25.8%	12	24.5%	44	25.4%		
	Doctors may lose some of their expertise if AI is used in their workflow	4	3.2%	2	4.1%	6	3.5%		
	Complexity of AI‎	2	1.6%	3	6.1%	5	2.9%		
	Lack of proper training	4	3.2%	2	4.1%	6	3.5%		
	Lack of Financial resources	1	0.8%	0	0.0%	1	0.6%		

ChatGPT was the most commonly used AI tool among respondents, followed by Google Assistant, Cortana, Quillbot, and Gemini.

The main obstacles to applying AI were patient data security concerns (64.2%), potential ethical and human implications of using AI in medicine (25.4%), and insufficient training (3.5%). Half feared losing their expertise, and AI had a difficult learning curve.

## DISCUSSION

Artificial intelligence refers to the capacity of computers and associated systems to do activities that often necessitate human cognitive abilities, including learning, reasoning, decision-making, comprehension, recognition, and natural language processing (NLP). Machine learning refers to the computer’s ability to understand data intelligently. When machine learning involves analysing images and videos, necessitating artificial neural networks, it’s Deep learning. The large language model is a deep learning algorithm that can analyse and understand text by learning from large amounts of data. ChatGPT4 from OpenAI, Llama from Meta, Claude from Anthropic, and Bard from Google are some of the language models available today. However, they have yet to be developed exclusively for health care. Machine learning, expert systems, speech recognition, planning, robotics, vision, and NLP processing are all considered subfields of artificial intelligence.^11^ AI-based algorithms are already implemented in many health products, including mobile applications and wearable devices.^12^

In rheumatology, a diagnosis is often established by evaluating a combination of clinical characteristics, lab reports, and radiograph findings. Artificial intelligence (AI) may assist with screening, diagnosis, monitoring, risk assessment, prognosis determination, attaining the best possible treatment outcome, de novo drug discovery, patient education, and counselling. It can also improve basic science research, increasing our understanding of the disease pathophysiology of rheumatic diseases. AI may help analyse large volumes of data like multiomics and play a role in identifying new biomarkers.^13–14^ AI has made tremendous achievements in the medical field over the past few years. It may assist rheumatologists in medical writing and provide a wealth of information to help produce high-quality articles. However, AI cannot replace the critical thinking experience of rheumatologists.^15^ Studies evaluating the role of AI-based quantification of pulmonary HRCT for evaluation of ILD(AIqpHRCT), predicting flares and clinical trial enrichment in lupus, identification of tophi in ultrasound imaging based on transfer learning and clinical practice, and detection of osteoarthritis are promising.^16–19^

Currently, ML and DL models have advanced to the point where they can automate the identification of patients with RA by utilising comparable parameters. Bai et al. conducted a recent study using an artificial neural network to detect individuals with RA reliably by combining patient demographic data and antibody profiles. Their model attained an AUROC of 0.95 and an F1 score of 0.916, indicating a significant level of accuracy.^20^

In our study, most of the participants (98.3%) were aware of AI, but only 45.7% were aware of subtypes like machine learning (ML), deep learning (DL), and generative. More than 80% of the participants believed AI would be helpful in medicine, mainly in medical education followed by patient care. However, most of them had not received formal training in AI. Nearly one-third of our respondents routinely used AI in regular practice. A similar study was conducted on 98 healthcare professionals from the NHS Trust in London. The study found that over 40% of the professionals did not know ML and DL, while 79% believed that AI would be crucial in healthcare.^21^ These findings align with our results. In another survey among Pakistani doctors and medical students, of 470 participants, 71.3% had a basic knowledge of AI, but only 35.3% knew about its subtypes.^22^

A survey among French paediatricians revealed that 90% comprehended “artificial intelligence.” In comparison, 65% reported an understanding of the term “machine learning” and 54% of “neural network,” whereas only 40% expressed familiarity with “deep learning”.^8^

Nowadays, medical practitioners have encountered AI solutions in various specialities. However, in our study, only a few have undergone formal training. More specialised training in AI in healthcare is currently needed. Therefore, clinicians may directly engage AI tools in their clinical practice without receiving prior teaching on the underlying ideas of algorithms, the development and evaluation of AI solutions, and the associated limitations and potential biases.

In the study among dermatologists, 69% expressed the belief that AI would have a transformative impact on the field of dermatology.^10^ In the Ooi et al. study, a majority (89%) of radiologists concurred that AI would have a transformative impact on their field of expertise.^23^

In our study, 20.2% had concerns about Ethical issues, and 11.6% believed it could lead to clinical mishaps. Only a few had a fear of career threats. However, most participants expressed positive outlooks regarding the potential of AI in supporting healthcare professionals. This finding was consistent with prior research demonstrating the successful application of AI in image analysis within radiology, pathology, and dermatology units. This application has been beneficial in facilitating diagnoses and lowering medical errors.

In the study conducted by Tezpal et al., A notable absence of knowledge about AI among the participants was identified, which can be attributed to the need for more formal education provided to healthcare practitioners. Regardless of the initial level of awareness, the majority of participants held the belief that AI has the potential to enhance population health outcomes.^24^

Undoubtedly, AI possesses advantages and disadvantages contingent upon the attitude and behaviour of healthcare professionals, which play a crucial role in progressing forward. Some perceive AI as a component of healthcare’s digital transformation, serving as clinical extenders and streamlining medical procedures. However, some believe that AI may harm the medical industry by altering the roles of physicians and other healthcare personnel.^25^

## LIMITATIONS

Our study had limitations. Firstly, due to the multiple distribution channels for online survey. Elderly rheumatologists are less active on social media compared to the residents. The residents may be more exposed to AI during their training. Secondly, there was an overlap of users between social media and email platforms, and we were not aware of how many would be using both. Hence, determining the response rate was difficult. However, we contacted an average of 1500 rheumatologists in the IRA (Indian Rheumatology Association) registry where most of the practising rheumatologists are members. Thirdly, the duration of the study was limited to three months. Fourthly, we could have received more responses if we had circulated offline forms, and getting offline forms filled out in a diverse country like India would be challenging.

## CONCLUSION

This study demonstrates a significant level of awareness but needs more knowledge about AI among Rheumatologists, which can be attributed to their lack of formal training in this field. The majority of participants held the idea that artificial intelligence (AI) has the potential to enhance health outcomes.

## AUTHOR CONTRIBUTIONS

All authors have accepted responsibility for the entire content of this manuscript and approved its submission. MM: research conception/design, data acquisition, data analysis/interpretation, manuscript preparation, final approval SM: research conception/design, data analysis/interpretation, manuscript preparation RB, AD, PM&VG: research conception/design, data acquisition, data analysis/interpretation.

## CONFLICT OF INTEREST

The authors declare no conflict of interest.

## RESEARCH FUNDING

None declared.

## ETHICAL APPROVAL AND CONSENT

The study was approved by the Institutional Ethical Committee of JSS Medical College, Mysore dated June 13,2024. (Ethical approval reference number JSSMC/IEC/130624/40NCT/2024-25). Since it involved an online survey, participant consent was obtained electronically before completing the questionnaire.

## AVAILABILITY OF DATA AND MATERIAL

The datasets used and analysed during the current study are available from the corresponding author on reasonable request.

Artificial Intelligence (AI)-Assisted Technology: No Artificial Intelligence (AI)-Assisted Technology was used in AI for designing, drafting, or editing this manuscript.

## APPENDIX

### Online Survey.

Artificial intelligence (AI), as a significant technological advancement, aims to simulate and automate human intelligence. However, the data on the role of Artificial Intelligence in Rheumatic diseases is lacking. We are a group of doctors who want to study the effects of KAP of artificial intelligence amongst rheumatology professionals. Therefore, we want both Rheumatology residents and consultants to take this survey.

This survey takes less than 5 minutes. Your responses are kept confidential and anonymous. We will analyse the data for publication in a medical journal, to discuss the KAP of artificial intelligence amongst rheumatology professionals. If you have further questions, please contact Dr. Mahabaleshwar Mamadapur MD, DM.(Rheumatologist), at mahabaleshwarm@jssuni.edu.in

### QUESSTIONNAIRE: DEMOGRAPHIC DATA

1. Where do you practice?
2. Which of the following best describes your area of practice?
  - Private/corporate hospital
  - Teaching hospitals in the private sector
  - Teaching hospitals in Govt sector
  - Non-teaching hospitals in Govt Sector
  - Central Institute
3. Rheumatology trainee Yes/No
4. Age Group
  <25 years/25–35 years/36–45 years/46–60 years / >60 years
5. Gender: Male/Female

### KNOWLEDGE:

Kindly give your views (Multiple options can be marked)

6. Are you aware of artificial intelligence (AI)? Yes/No
7. From where/whom did you get to know about AI
  - Social media
  - Colleagues or friends
  - Academic forum or articles
  - Other…….
8. Have you heard of AI terminologies like deep learning AI, machine learning AI, generative AI, etc?
  - Yes
  - No

### ATTITUDE

Kindly give your views (Multiple options can be marked)

9. Would AI be a useful tool in the medical specialty?
  - Yes
  - No
  - Maybe
  - neutral
10. What are the areas where you think AI and AI would be helpful in medical specialty?
  - Medical Education
  - Diagnosis and management of patients
  - Research and publication
  - Clerical work and documentation of patent records
  - Patient counselling
  - Other…
11. What aspect of medical education do you think AI will be useful? (Multiple answers)
  - Typing articles for publication
  - Preparing presentations
  - Providing reading materials for students
  - Exams and Evaluation
  - e. None of the above
  - f. Other
12. Do you think AI will promote better comprehension and learning of complex topics in medicine?
  - Yes
  - No
  - neutral
13. Should artificial intelligence be part of the medical curriculum?
  - Yes
  - No
  - neutral
14. Do you believe AI technology does threaten your career?
  - Yes
  - No
  - neutral
15. Do you believe that AI will provide ethical challenges?
  - Yes
  - No
  - neutral
16. Which of the following areas in clinical practice do you think AI may be of use
  - Clinical diagnosis and management
  - Patient counselling, issuing handouts, etc
  - Documentation and clerical work
  - Calculation of drug dosages, infusions, disease scores, etc
  - Interpretation of multiple laboratory reports and images
  - Develop personalized treatment solutions for patients.
  - Make personalized pharmacological prescriptions for individuals.
  - Other
17. What are the possible problems, AI may lead to in medical research and publication?
  - Ethically wrong
  - Authorship conflict
  - Misleading scientific information/statistical manipulation
  - Other…
18. What are the merits of AI according to you?
  - Ability to learn and refine its answers
  - Provide answers within a short timespan
  - Accuracy and relevancy of information
  - Other…
19. What do you think of the demerits of AI?
  - Not available in all languages
  - Can't personalize therapy
  - Ethical issues
  - May lead to a clinical mishap
  - Other…
20. What do you think about AI tools in the medical specialty in the future?
  - Unlikely to influence medical specialty
  - May influence/likely to stay as an optional use
  - A must in the future/part of the routine in medicine
  - Transform the field of medical specialty
  - Other…
21. Would you recommend AI to your colleagues in medicine
  - Yes
  - No

### PRACTICE

22. Have you attended webinars or panel discussions? on the role of AI in healthcare?
  - Yes
  - No
23. Have you had any formal sessions on using various AI tools?
  - Yes
  - No
24. Have you ever interacted with AI via particular or non-specific training?
  - Yes
  - No
25. Which of the following AI tools do you use regularly? (Multiple options)
  - ChatGPT
  - Google Assistant
  - Gemini Others
  - Quillbot
  - Cortana (Microsoft)
26. How often do you use AI?
  - Routinely
  - Rarely
27. What else do you use AI for?.......
28. What are the obstacles to the implementation of AI?
  - Patient data security
  - Threat to the ethical and human aspects of medicine
  - Doctors may lose some of their expertise if AI is used in their workflow.
  - Complexity of AI
  - Lack of proper training
  - Lack of financial resources
  - Lack of interest
  - Other:
